# A brief research report of suicide rates in the Brazilian elderly over a 12-year period: the lack of association of the "*Setembro Amarelo*" campaign for suicide prevention

**DOI:** 10.3389/fpsyt.2024.1354030

**Published:** 2024-07-25

**Authors:** Camila Corrêa Matias Pereira, Vahid Najafi Moghaddam Gilani, José Ignacio Nazif-Munoz

**Affiliations:** Service sur les dépendances, Faculté de médecine et des sciences de la santé, Université de Sherbrooke, Longueuil, QC, Canada

**Keywords:** suicide, suicide prevention, public health, aged, health campaign, public policy, mortality

## Abstract

**Objectives:**

Aiming to disseminate information related to suicide prevention in Brazil, the “*Setembro Amarelo*” campaign has been conducted since 2015. The objective of this study is to assess the association between this campaign and elderly suicide rates over a 12-year period.

**Methods:**

Data were gathered from the Mortality Information System and the Notifiable Diseases Information System, established by public institutions in Brazil. An interrupted time-series framework was applied to assess the association between the “*Setembro Amarelo*” campaign and suicide mortality rates in the elderly population (60 et plus) in the southeastern region of Brazil. We consider three monthly outcomes: all suicides, suicides in males and suicide in females. We operationalize the campaign assuming three effects: short-term, declining and sustained. The period of analysis was from 2011-2022.

**Results:**

The suicide-mortality rate over time has remained stable; the average rate in the pre-campaign period was 0.028 and increased slightly to 0.035. Regardless of the campaign’s operationalization and the outcome used, results show no significant associations between the campaign and elderly suicide rates. The campaign was associated with non-significant decreased effects of 15% (P=0.532) in the short term, and 16% (P=0.446) assuming the campaign was sustained.

**Conclusions:**

There is a lack of association between the campaign and suicide rates, among the elderly in Brazil’s southeastern region. As suicide is complex and multifactorial, more research is needed. The campaign, while raising awareness and reducing stigma, may not reduce suicides. To reduce the suicide rate in the elderly requires addressing social, economic and cultural factors, multisectoral interventions, and upholding basic human rights.

## 1 Introduction

The World Health Organization (WHO) underscores the alarming worldwide impact of suicide, with more than 700,000 deaths annually. Suicide prevention prominently features on the WHO’s agenda and is integrated as an indicator in the United Nations’ Sustainable Development Goals (1). Effective suicide prevention strategies require understanding of risk and protective factors across different life stages (2). Elderly individuals face the highest global suicide risk, with elevated lethality (3–6). Unique risk factors for this group include among others social isolation, bereavement, loss of social roles, debilitating illnesses, depression, loneliness, and access to lethal means (items that can be used in a suicide, such as firearms and certain drugs or toxic substances) (3–10). In general elderly individuals exhibit a heightened suicide rate, attributed to more lethal attempts (11, 12).To address prevention, there is an urgent need for comprehensive suicide prevention programs (SPP) involving diverse sectors such as health, education, social welfare, media and case notification, aiming to optimize tailored initiatives across contexts (2, 13, 14).

The rapid aging of the population underscores a pressing issue regarding the deteriorating epidemiological circumstances for older adults, as the provision of care, aid, and backing for this demographic fails to advance adequately in terms of speed, urgency, or scope. Among the specific challenges encountered by this demographic, especially in low and middle-income countries, is the prevalence of suicide (15–18). While the World Health Organization (WHO) marks the onset of old age at 65, as this may be indicative of potential retirement and eligibility for certain benefits, this age threshold may drop to 60 in areas with shorter life expectancies (19, 20). In Brazil, its Federal Law No. 8.842/1994, classifies individuals as elderly if they are 60 years of age or older (21). The suicide death rates of elderly in Brazil from 1996 to 2018 have increased by 162.2% (age group 60-69), 141.4% (70–79), and 189.3% (80 years and above) respectively (11). Overall, all Brazilian regions have experienced an increase of suicide rates in older adulthood from 1996 to 2018, with notable percentages in the North (81.1%), Northeast (126.5%), Southeast (26.6%), Central-West (28.5%) and South (17.8%) (11).

Studies conducted in Brazil have identified that the most frequent social reasons triggering suicide among the elderly include financial problems, unemployment, employment instability (e.g., farming, mining), relationship difficulties, family conflicts, social isolation, and loneliness (22–26). In general, the prevalence of male suicides is higher, associated with the fact that men are more competitive, impulsive, and have easier access to lethal means (27). Additionally, societal expectations within the Brazilian patriarchal culture, which cast men in the role of the primary provider for the family, further contribute to this phenomenon (27). Also, retirement, social exclusion and deprivation of social support, very common in the reality of the elderly, are linked to preliminary depression diagnoses and demoralization, important risk factors for suicide (28–30). In Brazil, there are scientific publications addressing aging and suicide in general; however, few focus on elderly suicide mortality, underscoring the need for further research on these topics within the country (4, 31–33).

In Brazil, in 2015, an initiative led by the Center for Valorization of Life (CVV- Brazilian helpline), the Federal Medical Council, and the Brazilian Psychiatric Association created a campaign called “*Setembro Amarelo*,” (Yellow September), aimed at suicide prevention and raising awareness. While the campaign runs throughout the year, its concentrated events occur in September. Throughout this month, the campaign endeavors to raise societal awareness and disseminate information related to suicide prevention in the country. This includes organizing events to discuss mental health and suicide prevention, reducing stigma, sharing data on suicide rates, promoting help channels, distributing educational materials, providing specific training, and lighting national landmarks in yellow (34–36). A limited number of studies from Brazil (37–40) have endeavored to evaluate changes following the campaign’s implementation. The impact of this campaign on suicide mortality rates, in this specific age-group, remains unexamined. The present study investigated Brazilian elderly suicide rates over a 12-year period, considering the launch and implementation of the “*Setembro Amarelo*” campaign.

## 2 Methods

### 2.1 Study design

We applied a quasi-experimental design by using an interrupted time-series (ITS) framework (41) spanning twelve years (2011 to 2022). We specifically assess the association between the “*Setembro Amarelo*” campaign and suicide mortality rates in the elderly population. We selected three outcomes: i) all suicides, ii) suicides in males and iii) suicide in females.

### 2.2 Data

We utilized publicly available and official information derived from the Brazilian Government. Data were extracted from the Mortality Information System (SIM) and the Notifiable Diseases Information System (SINAN), both established by the Brazilian Ministry of Health and accessible via the DATASUS online platform. This system functions through the systematic compilation of data concerning vital statistics, encompassing mortality and survival rates, as well as epidemiological and morbidity insights. We restricted our analysis to monthly suicide mortality rates for the southeastern region of Brazil, which encompasses São Paulo, Rio de Janeiro, Minas Gerais, and Espírito Santo. This dataset covers the years from 2011 to 2022. The database (case registration) for the SIM and SINAN was more complete in the southeast region, therefore, this region was chosen for the analyzed years. Additionally, we included data on unemployment rates obtained from the International Labour Organization (ILO) for the same period, 2011 to 2022.

### 2.3 Study variables

#### 2.3.1 Dependent variables

Counts of suicide mortality rates in the elderly population (Yij) and counts of suicides for male (YMij) and female (YWij) respectively, were derived from the total number of fatal self-inflicted injuries of individuals of 60 years and over, information gathered from the Brazilian Ministry of Health. We have included the classification according to the International Statistical Classification of Diseases and Related Health Problems 10th Revision (ICD-10) (42), including ICD codes (X60 to X84), corresponding to Intentional self-harm. The rate was obtained by dividing the total number of individuals aged 60 and more, population numbers were gathered from the Brazilian census data.

#### 2.3.2 Policy time-varying covariates

We operationalized “*Setembro Amarelo*” campaign, in three forms: i) *Short-term effect*: 0 for the years 2011 to 2014, 0 for January to August and for October to December for the years 2015 to 2022, and 1 for September for the years 2015 to 2022. We assumed this campaign was effective only during September since 2015; ii) *Declining effect*: 0 for the years 2011 to 2014, 0 for January to August and for December for the years 2015 to 2022, and since 2015 1, for Sep, 0.5 for October, and 0.25 for November. We assume this campaign was effective from September to November however its effect may have declined at the third month of implementation; iii) *Sustained effect*: 0 for the years 2011 to 2014, 0 for January to August and for December for the years 2015 to 2022, and since 2015 1 for the months of September, October, and November.

#### 2.3.3 Control variables

In our study, we addressed the potential impact of seasonal variations by incorporating Fourier terms, using pairs of sine and cosine functions to model these patterns. Additionally, unemployment rates were included as a control variable. Specifically, these rates pertain to adults aged 60 years and older.

### 2.4 Statistical analysis

An extension of generalized linear models was employed, incorporating both the Poisson and Negative Binomial models. These models are well suited for the analysis of count data, such as number of suicides. The Poisson model is a reliable choice for modeling count data when events occur at a consistent rate over time. On the other hand, the Negative Binomial model offers flexibility and accounts for data over-dispersion, which is common when dealing with rare events like suicides. The use of the Poisson and Negative Binomial models within the ITS analysis ensures a thorough exploration of temporal patterns and trends, providing more nuanced insights into the connection between the campaign and suicide rates.

## 3 Results

**Figure 1:**
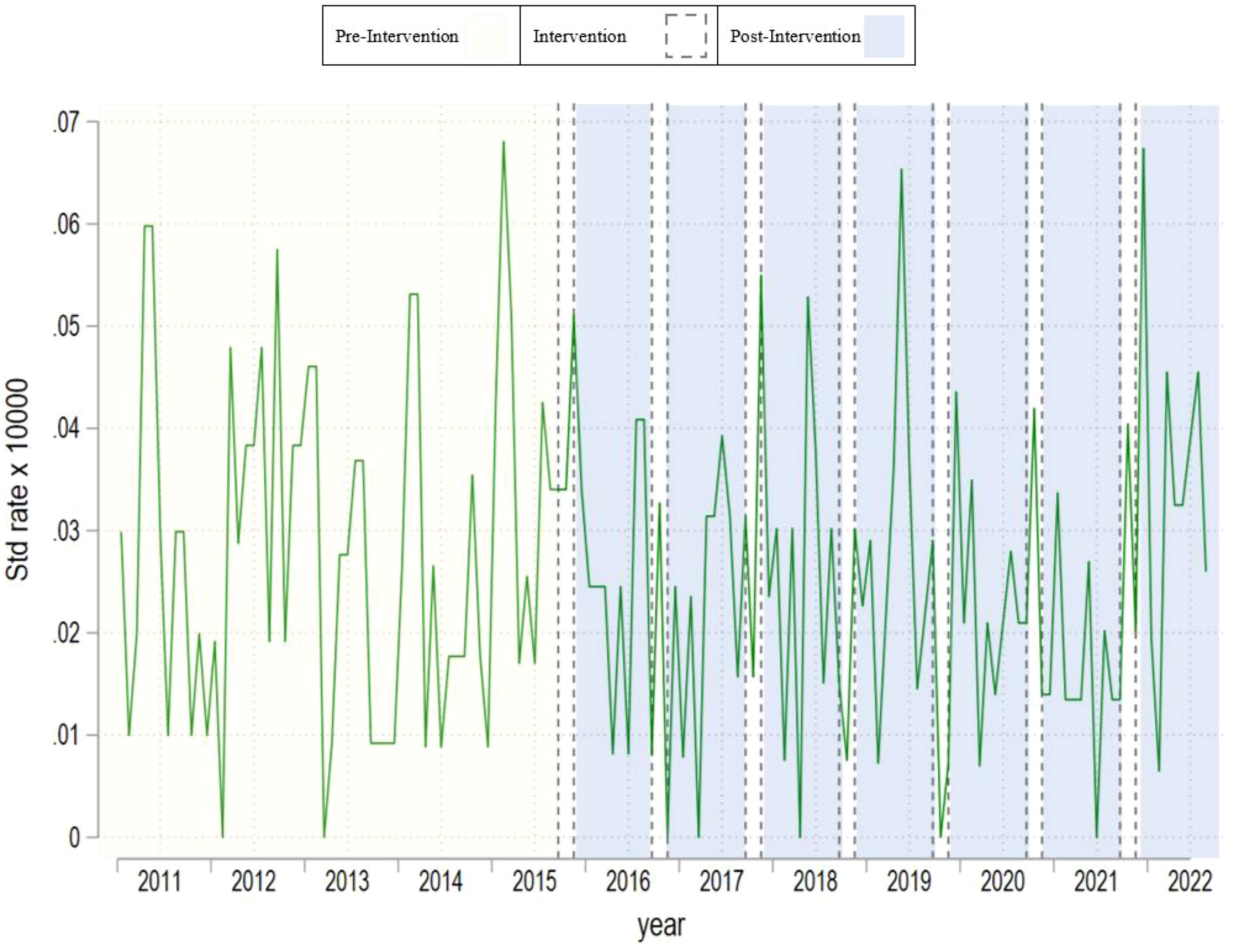
Monthly time–series plot for suicide rate of all individuals aged above 60 years old.

**Figure 2:**
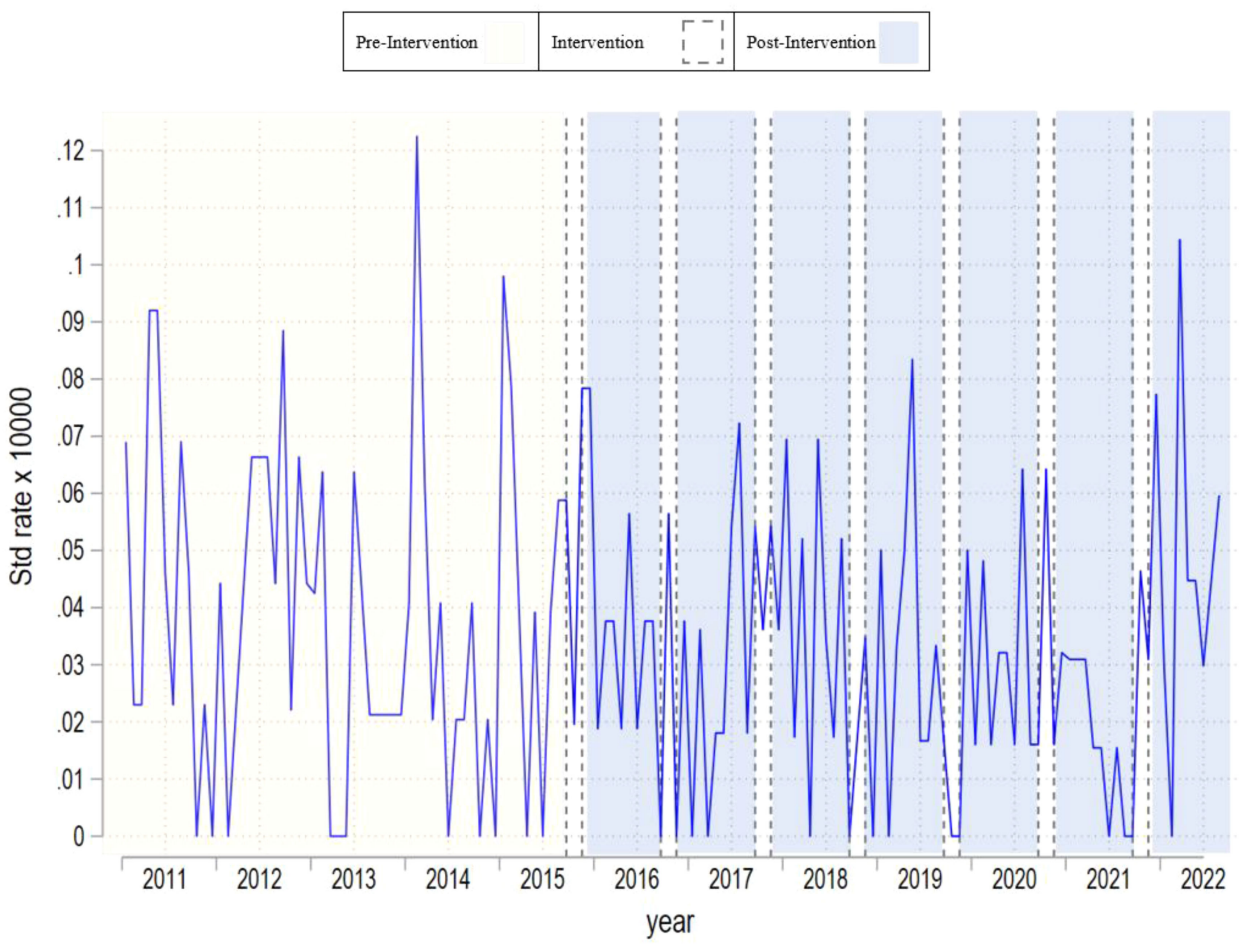
Monthly time–series plot for suicide rate of males aged above 60 years old.

**Figure 3:**
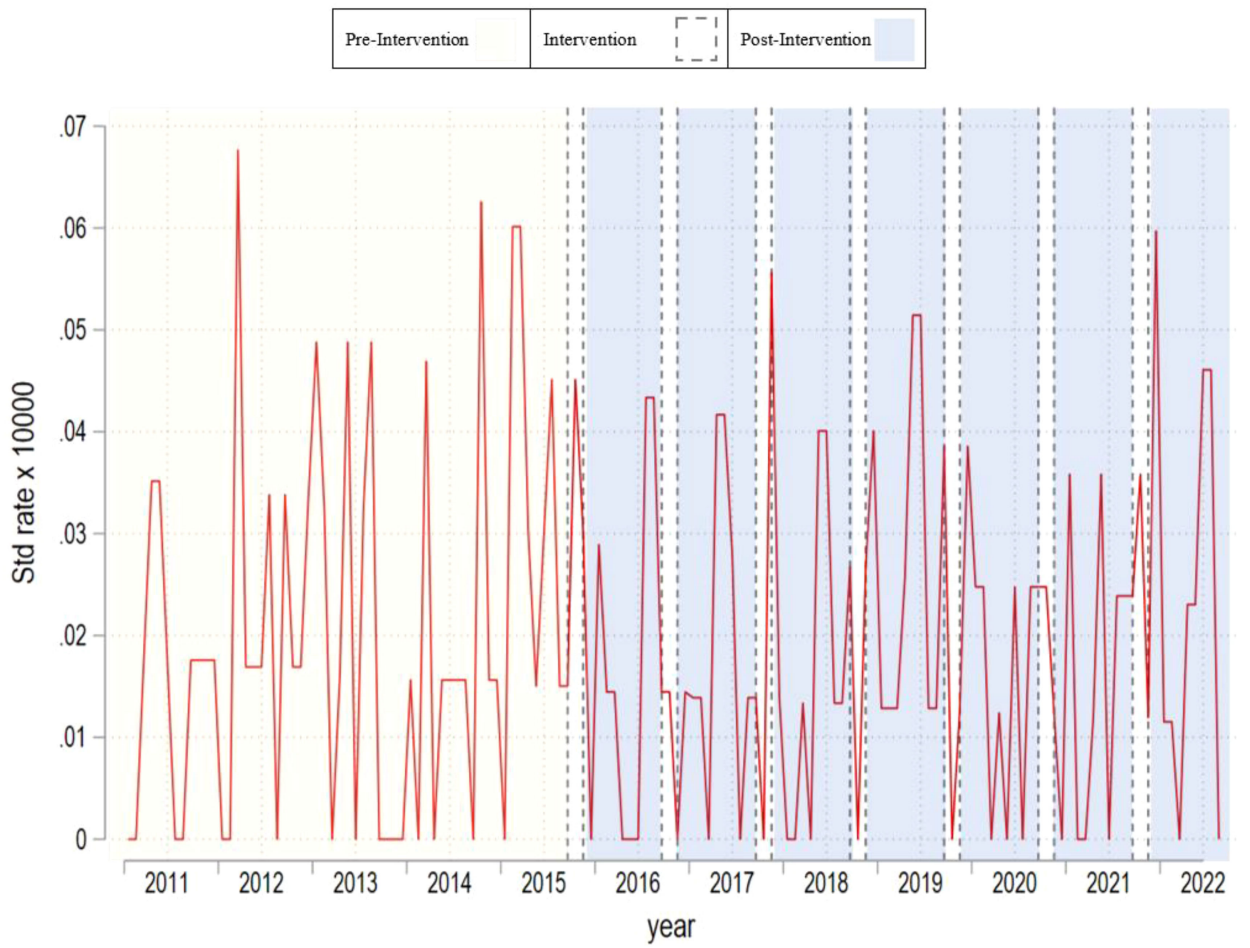
Monthly time–series plot for suicide rate of females aged above 60 years old.

**Table 1** provides an overview of summary statistics regarding suicide mortality rates across all gender categories. **Figures 1**–**3** present monthly time-series plots for the suicide mortality rates: all individuals, males, and females aged above 60 years old, respectively. **Tables 2**, **3** provide the results Poisson and the Negative Binomial models.

**Table 1:** Monthly suicide rates per 100 000 population before, during and after three operationalizations of the “*Setembro Amarelo*” Campaign.

Period	Suicide rate (All)			Suicide rate (Male)			Suicide rate (Female)		
	Mean	Minimum	Maximum	Mean	Minimum	Maximum	Mean	Minimum	Maximum
Short-term effect									
Pre-Intervention	0.028	0.000	0.068	0.038	0.000	0.122	0.020	0.000	0.068
Intervention 1: Sep 2015	0.034	0.034	0.034	0.059	0.059	0.059	0.015	0.015	0.015
Post Intervention Year 1	0.029	0.008	0.051	0.040	0.019	0.078	0.020	0.000	0.045
Intervention 2: Sep 2016	0.008	0.008	0.008	0.000	0.000	0.000	0.014	0.014	0.014
Post Intervention Year 2	0.022	0.000	0.039	0.028	0.000	0.072	0.017	0.000	0.042
Intervention 3: Sep 2017	0.031	0.031	0.031	0.054	0.054	0.054	0.014	0.014	0.014
Post Intervention Year 3	0.027	0.000	0.055	0.040	0.000	0.069	0.017	0.000	0.056
Intervention 4: Sep 2018	0.015	0.015	0.015	0.000	0.000	0.000	0.027	0.027	0.027
Post Intervention Year 4	0.027	0.007	0.065	0.031	0.000	0.083	0.024	0.000	0.051
Intervention 5: Sep 2019	0.029	0.029	0.029	0.017	0.017	0.017	0.039	0.039	0.039
Post Intervention Year 5	0.020	0.000	0.044	0.026	0.000	0.064	0.015	0.000	0.039
Intervention 6: Sep 2020	0.021	0.021	0.021	0.016	0.016	0.016	0.025	0.025	0.025
Post Intervention Year 6	0.019	0.000	0.042	0.023	0.000	0.064	0.018	0.000	0.039
Intervention 7: Sep 2021	0.013	0.013	0.013	0.000	0.000	0.000	0.024	0.024	0.024
Post Intervention year7	0.034	0.006	0.067	0.047	0.000	0.104	0.024	0.000	0.060
Declining effect or Sustained effect									
Pre-Intervention	0.028	0.000	0.068	0.038	0.000	0.122	0.020	0.000	0.068
Intervention 1: Sep, Oct, Nov 2015	0.040	0.034	0.051	0.052	0.020	0.078	0.030	0.015	0.045
Post Intervention Year 1	0.026	0.008	0.041	0.038	0.019	0.078	0.016	0.000	0.043
Intervention 2: Sep, Oct, Nov 2016	0.014	0.000	0.033	0.019	0.000	0.056	0.010	0.000	0.014
Post Intervention Year 2	0.023	0.000	0.039	0.028	0.000	0.072	0.019	0.000	0.042
Intervention 3: Sep, Oct, Nov 2017	0.034	0.016	0.055	0.048	0.036	0.054	0.023	0.000	0.056
Post Intervention Year 3	0.025	0.000	0.053	0.025	0.000	0.053	0.015	0.000	0.040
Intervention 4: Sep, Oct, Nov 2018	0.018	0.008	0.030	0.017	0.000	0.035	0.017	0.000	0.035
Post Intervention Year 4	0.028	0.007	0.065	0.032	0.000	0.083	0.026	0.013	0.051
Intervention 5: Sep, Oct, Nov 2019	0.012	0.000	0.029	0.006	0.000	0.017	0.017	0.000	0.039
Post Intervention Year 5	0.023	0.007	0.044	0.032	0.016	0.064	0.017	0.000	0.039
Intervention 6: Sep, Oct, Nov 2020	0.026	0.014	0.042	0.032	0.016	0.064	0.021	0.012	0.025
Post Intervention Year 6	0.017	0.000	0.034	0.019	0.000	0.032	0.015	0.000	0.036
Intervention 7: Sep, Oct, Nov 2021	0.025	0.013	0.040	0.026	0.000	0.046	0.024	0.012	0.036
Post Intervention Year 7	0.035	0.006	0.067	0.048	0.000	0.104	0.025	0.000	0.060

**Table 2:** Poisson model results for suicide rate of all types of gender groups.

Period	Suicide rate (All)			Suicide rate (Male)			Suicide rate (Female)		
	IRR	95%CI	P-Value	IRR	95%CI	P-Value	IRR	95%CI	P-Value
Short-term effect	0.85	0.53 1.38	0.532	0.62	0.29 1.30	0.212	1.18	0.64 2.16	0.588
Unemployment rate	0.85	0.72 1.02	0.091	0.94	0.78 1.12	0.522	0.80	0.62 1.02	0.075
Time	1.00	0.99 1.00	0.315	0.99	0.99 1.00	0.770	1.00	0.99 1.01	0.103
Seasonal Trend (cos)	0.94	0.82 1.08	0.420	0.98	0.82 1.17	0.892	0.88	0.72 1.09	0.264
Seasonal Trend (sin)	1.02	0.88 1.19	0.729	1.05	0.86 1.27	0.606	0.98	0.77 1.25	0.907
MLE	-282.83			-234.99			-210.40		
AIC	4.09			3.41			3.06		
BIC	-501.84			-502.44			-499.93		
Declining effect	1.02	0.92 1.14	0.606	0.82	0.56 1.20	0.318	1.00	0.67 1.49	0.996
Unemployment rate	1.30	1.28 1.32	0.000	0.93	0.77 1.11	0.432	0.80	0.63 1.02	0.077
Time	0.99	0.99 0.99	0.000	0.99	0.99 1.00	0.894	1.00	0.99 1.01	0.103
Seasonal Trend (cos)	0.93	0.81 1.09	0.396	0.98	0.81 1.19	0.878	.87	0.69 1.09	0.245
Seasonal Trend (sin)	1.04	0.87 1.26	0.607	1.09	0.87 1.37	0.440	.97	0.73 1.29	0.874
MLE		-213.18		-235.64			-210.55		
AIC		3.10		3.42			3.06		
BIC		-641.13		-501.13			-499.63		
Sustained effect	0.84	0.54 1.30	0.446	0.67	0.36 1.23	0.197	1.07	0.60 1.92	0.808
Unemployment rate	0.85	0.71 1.02	0.082	0.93	0.78 1.11	0.467	0.80	0.63 1.02	0.078
Time	1.00	0.99 1.00	0.281	0.99	0.99 1.00	0.868	1.00	0.99 1.01	0.107
Seasonal Trend (cos)	0.94	0.81 1.08	0.420	0.99	0.83 1.19	0.981	0.87	0.71 1.07	0.212
Seasonal Trend (sin)	1.03	0.87 1.23	0.682	1.05	0.85 1.31	0.623	1.01	0.76 1.31	0.992
MLE		-282.72			-235.23	-210.52			
AIC		4.09			3.41	3.06			
BIC		-502.06			-501.96	-499.69			

**Table 3:** Negative binomial model results for suicide rate of all types of gender groups.

Period	Suicide rate (All)			Suicide rate (Male)			Suicide rate (Female)		
	IRR	95%CI	P-Value	IRR	95%CI	P-Value	IRR	95%CI	P-Value
Short-term effect	1.03	0.51 1.56	0.901	0.59	0.00 1.37	0.309	1.59	0.72 2.11	0.248
Unemployment rate	0.82	0.64 1.00	0.040	0.92	0.74 1.10	0.423	0.76	0.52 0.99	0.045
Time	1.00	0.99 1.00	0.206	0.99	0.99 1.00	0.863	1.00	0.99 1.01	0.079
Seasonal Trend (cos)	0.92	0.79 1.05	0.223	0.98	0.81 1.15	0.797	0.84	0.65 1.04	0.135
Seasonal Trend (sin)	1.06	0.92 1.22	0.381	0.93	0.88 1.25	0.495	1.03	0.81 1.26	0.767
MLE	-280.76			-234.26			-207.53		
χ²	0.37			0.50			0.23		
Declining effect	1.12	0.71 1.53	0.562	1.03	0.49 1.57	0.906	1.16	0.57 1.77	0.582
Unemployment rate	0.81	0.63 0.99	0.034	0.91	0.74 1.09	0.346	0.76	0.52 1.00	0.050
Time	1.00	0.99 1.00	0.220	0.99	0.99 1.00	0.875	1.00	0.99 1.00	0.093
Seasonal Trend (cos)	0.89	0.74 1.04	0.179	0.97	0.77 1.16	0.748	0.83	0.61 1.04	0.116
Seasonal Trend (sin)	1.09	0.82 1.27	0.290	1.11	0.88 1.25	0.335	1.03	0.77 1.30	0.811
MLE	-280.60			-234.81			-208.01		
χ²	0.34			0.63			0.31		
Sustained effect	1.12	0.57 1.68	0.660	0.81	0.06 1.57	0.636	1.39	0.62 2.18	0.321
Unemployment rate	0.81	0.63 0.99	0.036	0.92	0.74 1.10	0.389	0.75	0.51 0.99	0.045
Time	1.00	0.99 1.00	0.205	0.99	0.99 1.00	0.898	1.00	0.99 1.00	0.089
Seasonal Trend (cos)	0.91	0.77 1.04	0.198	0.98	0.81 1.16	0.862	0.83	0.63 1.03	0.096
Seasonal Trend (sin)	1.08	0.91 1.25	0.326	1.07	0.89 1.25	0.496	1.05	0.79 1.31	0.658
MLE		-280.68		-234.70			-207.68		
χ²		0.35		0.61			0.25		

In **Table 1**, we observe that suicide mortality rates over time are relatively stable. Whereas the mean in the pre campaign period is 0.028 at the end of studied period the rate is slightly higher with 0.035. This trend is also observed in men and women respectively. Nevertheless, we observe differences across men and women rates. In the per period campaign men’s rates were 0.038 and women’s 0.020, and at the end of the analyzed period these rates were 0.048 and 0.024 respectively. Based on the results of **Tables 2**, **3**, we fail to observe significant associations between the campaign and suicide mortality rates regardless of the campaign’s operationalization and the outcome used. The decision to collapse the data for the declining and sustained effects was made due to their similar statistical outcomes in preliminary analyses, simplifying the presentation and focusing on the most significant findings. Results were robust after we included unemployment rates as well.

Our analysis of the association between unemployment rates and suicide rates showed variability by the model and demographic group. In the Poisson model, unemployment rates were not significantly associated with changes in suicide rates. However, the Negative Binomial model indicated a significant impact for all individuals and females, but not for males, suggesting differing economic effects across demographic groups. Statistical tests for stationarity confirmed the reliability of these findings. The Augmented Dickey-Fuller (ADF) tests showed that the series are stationary, and the Kwiatkowski-Phillips-Schmidt-Shin (KPSS) tests indicated a presence of a stochastic trend at zero lags. These tests are detailed in the newly added **Table 4**, enhancing our understanding of the data’s characteristics and the robustness of our analysis.

**Table 4:** Results of Augmented Dickey-Fuller (ADF) and Kwiatkowski-Phillips-Schmidt-Shin (KPSS) Tests.

Suicide rate	Augmented Dickey-Fuller (ADF)				Lag order of Kwiatkowski-Phillips-Schmidt-Shin (KPSS)				
	Test Statistic	1%	5%	10%	0	1	2	3	4
Male	-7.620	-3.497	-2.887	-2.557	0.0437	0.0400	0.0383	0.0397	0.0411
Female	-7.620	-3.497	-2.887	-2.557	0.0488	0.0469	0.0443	0.0432	0.0425
Total	-7.620	-3.497	-2.887	-2.557	0.0367	0.0360	0.0394	0.0431	0.0448

## 4 Discussion

In this quasi-experimental study, we observe no significant differences between three different operationalizations of the “*Setembro Amarelo*” campaign and suicide mortality rates in the elderly population in four Brazilian States: São Paulo, Rio de Janeiro, Minas Gerais, and Espírito Santo. This lack of association is observed regardless of the operationalization of the campaign and statistical method applied. Our analyses also indicate that the campaign was not necessarily effective when targeting men nor women specifically. While the World Health Organization recommends conducting awareness and prevention campaigns utilizing mass media to promote mental health, raise awareness, reduce mental health stigma, and disseminate information about support resources, these efforts should be accompanied with specific prevention interventions (6, 43). Indeed, sparsely evaluated outcomes of suicide prevention campaigns have demonstrated contradictory and inconsistent results (43, 44), with positive effects on awareness and help-seeking (43–45), or the need for caution when developing strategies because of prejudice, misinformation and questioning how to reach vulnerable populations not considered in more consistent prevention efforts (44, 46).

Our study partially aligns with at least three studies developed in Brazil. First, a time-series study analyzed the temporal pattern of suicide mortality in the state of Ceará between 2009 and 2019. The study observed that the population aged 60 to 79 experienced a stabilization in suicide rates, raising questions about the campaign’s potential impact in this age group within this state (38). A second study conducted to identify changes following the implementation of “*Setembro Amarelo*”, in the Brazilian population, revealed that suicide-related incidence rates showed an upward trend after the program’s implementation. The suicide rate increased by 66.6% (37). Third, an interrupted time series study analyzed the evolution of elderly suicide rates in Brazil between 2011 and 2019 (30), observing a 14.3% increase in suicide rates after the campaign was implemented. These results raised considerable questions as to whether this increment was due to an adverse effect of the campaign’s implementation or a result of more effective case reporting (37).

The following elements are necessary to interpret our results. First, while there are guidelines issued by the Brazilian Psychiatric Association (47), based on international guidelines, on how to safely communicate or address suicidal behavior, many recommendations may not be followed. More specifically, the campaign may lack multi-sectoral actions, establishing an assistance network, implementing longitudinal strategies for monitoring at-risk groups (i.e. men, or elderly), as well as offering training and professional development opportunities (39). Relatedly, in September, the Brazilian campaign may lead many to discuss the topic of suicide, however, taking preventive actions and ensuring secure communication about suicide requires a more nuanced approach to avoid the risk of contagion (38, 48).

Second, suicide is generally underreported more than other causes of death, a trend that intensifies when concerning the elderly (2, 49–51). Death certificate data may be underreported due to various factors, including differing professional perspectives and training regarding their completion. Suspected suicide cases may be recorded as accidental poisonings or other external causes of mortality. Numerous taboos (for religious, cultural, or other reasons) and bureaucratic challenges, such as idealized post-mortem perceptions, life insurance implications, and the need for accurate cause of death, contribute to this context. Overcoming underreporting is crucial to enable reliable epidemiological analyses and, consequently, to inform effective care strategies (4, 52, 53). Two Brazilian studies analyzing suicide notifications after the campaign found no changes regarding self-harm case notifications; it may have even triggered the opposite effect as increases in cases in October compared to August were observed (37, 40).

Third, public campaigns aimed at preventing suicide among the elderly effectively should include widespread societal efforts on mental health and suicide awareness, information about help seeking, investing in access policies to mental health professionals and therapists, proactive engagement with psychiatric patients following discharge or a suicidal crisis, integration with primary health care services, fiscal support from professionals, social cohesion via social media, targeted online discussion forums for the elderly, telephone helplines, community integration interventions to combat loneliness, and public health messages emphasizing the importance of social involvement for all older individuals (4, 54–57). Particular attention to reducing stigma in the elderly should be considered in these campaigns. Indeed, the elderly often do not recognize or seek help for mental illness due to stigma, seeing it as a weakness and fearing the loss of independence. Tackling stigma needs focused efforts through professional training, public education, media collaboration, and inclusion in health and social care programs to encourage help-seeking and community integration (58, 59). Much of these elements have not been considered in the “*Setembro Amarelo*” campaigns and therefore next versions of this campaign should discern some of these strategies to target elderly more properly (47, 48).

Fourth, in addition to the need for interventions tailored to different life stages, it is also crucial to develop gender-specific interventions. Studies indicate that women are more likely to seek healthcare services, exhibit better coping with stigma, prejudice, and taboos compared to men, resulting in increased access to mental health services for this population (60, 61). Furthermore, social roles, such as being mothers and grandmothers, are often viewed differently for men. Men, traditionally seen as providers, tend to lose this role as they age. The loss of this social role represents a significant risk factor for suicide in the elderly, that is why socioeconomic issues, including economic crises, unemployment, and reductions in personal income, are significant risk factors, especially among men (33, 60, 61). Studies conducted in Brazil corroborate the international literature, as it is believed that men exhibit more competitive and impulsive behaviors than women, along with higher substance abuse rates, including alcohol and drug consumption (4, 62).

Last, suicide, in Brazil as a social phenomenon, must account for religious practices (63). In this country, most individuals claim a religious affiliation and consider religion a significant aspect of their existence (64, 65). The relationship between religion and suicidal behavior is complex and it needs more research in different cultures and religious backgrounds, as it can offer both protective factors, (e.g. coping mechanism and support system) and risk elements (e.g. assigning all life responsibility to God, blaming spiritual beliefs for failures when feeling forsaken, and interpreting stressors as either divine retribution or the influence of “evil forces.”) (66–70). For the elderly, spirituality and religiosity have been linked to improved quality of life and mental health promotion promising valuable contributions to geriatric psychiatry effective interventions and campaigns (71, 72). The extent to which “*Setembro Amarelo*” should consider religious aspects in its messages is something that should be studied further.

Our study should consider the following limitations to better temper the implication of its results. First, a large range of suicide risk factors and regional inequalities play significant roles in Brazil (33). Indeed, this country is characterized by multiple cultures and deep social inequalities, emphasizing that our findings cannot be generalized to the entire nation. It may be the case that in other regions, the campaign was effective not only in the elderly population but in others too. Our results suggest thus the identification of region-specific public policies to properly assess the extent to which this campaign may have not been effective (33). Secondly, to analyze suicide mortality rates and the “*Setembro Amarelo*” campaign, it is essential to consider case notification challenges, a prevalent issue in several countries, including Brazil, where underreporting in official records maybe prevalent (4, 38). Suicide statistics result from a complex process involving various stages, including reports from family members, witnesses, physicians, law enforcement, coroners, and statisticians. Due to these procedural intricacies, data may be distorted throughout, particularly in regions where social, economic, cultural, and religious factors contribute to the stigma surrounding suicidal behavior (38, 52). Third, while we used different operationalizations to understand the potential impact of the campaign, our application considered the overall post period after the campaign was implemented. So, we did not study if there were specific years in which the campaign could have been more effective. Last, while suicide is a multifactorial phenomenon, our study only covered specific results in terms of sex, and some models also included the variable unemployment—which was robust to our main results. As this is the first study in the elderly population, and no significant associations were found in this region, one potential explanation could be a more consistent network of social support and better access to health services for the elderly. However, this should be tested with other variables, such as family composition, state programs, and health services designed to support this population. Nevertheless, other factors such as socioeconomic status or marital status, which have been consistently associated with suicide outcomes were not considered and therefore other analyses are necessary to better understand the potential impact of this campaign in other subgroups of the elderly population in Brazil.

## 5 Conclusion

To our knowledge, this is the first study to assess the impact of the “*Setembro Amarelo*” campaign on suicide rates among the elderly population in Brazil’s Southeast region. Regardless of the campaign’s operationalization and the outcome used, no significant variation was observed. It is important to emphasize that suicide is a complex and multifactorial phenomenon. Therefore, the campaign alone, which aims to raise awareness and reduce stigma related to mental health promotion and suicide prevention, requires other actions to effectively tackle suicides at the population level.

We emphasize the importance of developing more scientific research and public policies for suicide prevention in Brazil, based on scientific knowledge, culturally adapted, focused on different stages of the life cycle, as well as sex and gender-specific interventions. Public policies, regardless of their format, need to be evaluated and planned to achieve continuous interventions, not limited to specific campaign months, as suicide is a public health issue that occurs daily in Brazil and around the world.

## Data availability statement

The datasets presented in this study can be found in online repositories. The names of the repository/repositories and accession number(s) can be found below: http://tabnet.datasus.gov.br/cgi/tabcgi.exe?sih/cnv/fruf.def Official morbimortality data in Brazil are made available by DATASUS - Information Technology Department of the Brazilian Public Health Care System (SUS).

## Author contributions

CC: Conceptualization, Investigation, Methodology, Writing – original draft, Writing – review & editing, Data curation, Visualization. VN: Data curation, Formal analysis, Investigation, Methodology, Software, Writing – original draft, Writing – review & editing. JN: Conceptualization, Formal analysis, Funding acquisition, Investigation, Methodology, Project administration, Resources, Supervision, Visualization, Writing – original draft, Writing – review & editing.
